# Identifying Positioned Nucleosomes with Epigenetic Marks in Human from ChIP-Seq

**DOI:** 10.1186/1471-2164-9-537

**Published:** 2008-11-13

**Authors:** Yong Zhang, Hyunjin Shin, Jun S Song, Ying Lei, X Shirley Liu

**Affiliations:** 1 Department of Biostatistics and Computational Biology, Dana-Farber Cancer Institute, Harvard School of Public Health, 44 Binney St, Boston, MA 02115, USA; 2 Simons Center for Systems Biology, Institute for Advanced Study, Einstein Dr, Princeton, NJ 08540, USA

## Abstract

**Background:**

*In vivo *positioning and covalent modifications of nucleosomes play an important role in epigenetic regulation, but genome-wide studies of positioned nucleosomes and their modifications in human still remain limited.

**Results:**

This paper describes a novel computational framework to efficiently identify positioned nucleosomes and their histone modification profiles from nucleosome-resolution histone modification ChIP-Seq data. We applied the algorithm to histone methylation ChIP-Seq data in human CD4^+ ^T cells and identified over 438,000 positioned nucleosomes, which appear predominantly at functionally important regions such as genes, promoters, DNase I hypersensitive regions, and transcription factor binding sites. Our analysis shows the identified nucleosomes play a key role in epigenetic gene regulation within those functionally important regions via their positioning and histone modifications.

**Conclusion:**

Our method provides an effective framework for studying nucleosome positioning and epigenetic marks in mammalian genomes. The algorithm is open source and available at .

## Background

Chromatin structure widely manifests itself in various aspects of mammalian development and disease. The key structural element of chromatin is the nucleosome, which consists of an octameric histone core wrapped by 146 bps of DNA [1]. Nucleosomes play two major roles in epigenetic regulation of gene expression. The first is to limit DNA accessibility to cellular machinery [2-5] through specific positioning of nucleosome core particles, which can be remodeled in an ATP-dependent manner. The second is to regulate transcriptional activities through covalent modifications (e.g. methylation, acetylation and phosphorylation) of the tails of four core histone types H2A, H2B, H3 and H4 [6-9]. Therefore, characterizing the global locations and modification marks of positioned nucleosomes is a crucial step towards unraveling the mechanism of epigenetic regulation in eukaryotes.

High-throughput mapping of positioned nucleosomes has been conducted in yeast [10,11] and selected human promoters [12] using high resolution tiling microarrays. Several studies have also profiled genome-scale histone modification marks using Chromatin ImmunoPrecipitation (ChIP) coupled with microarrays (ChIP-chip) [8,13-15], although only one study in yeast [7] mapped the modifications at nucleosome resolution. Recent developments in high-throughput sequencing techniques offer a promising alternative to microarrays in analyzing genome-wide nucleosome positioning and histone modifications. Barski *et al. *[16] used Solexa to conduct nucleosome-resolution ChIP-Seq of twenty different histone methylation marks as well as H2A.Z in human CD4^+ ^T cells, although the analysis in that study was performed at a much lower resolution than offered by the data. Recently, the same group also sequenced MNase-digested nucleosomes and performed ChIP-Seq of eighteen histone acetylation marks [17,18]. As an extension of their histone methylation study, they attempted to reveal the effects of nucleosome positioning [17] and combinatorial patterns of histone modifications on gene activity [18] separately. However, genome-wide studies of positioned nucleosomes coupled with histone modifications in human still remain limited.

We thus developed a novel computational framework for analyzing histone modification ChIP-Seq data at nucleosome resolution in a global scale. By combining the sequenced tags from all histone methylation ChIP-Seq data [16] and employing signal processing techniques, we comprehensively identified modified positioned nucleosomes in functionally important regions and revealed their distribution throughout the human genome. Through subsequent statistical modeling, we were then able to assign histone methylation marks to each positioned nucleosome in CD4^+ ^T cells, revealing the key roles of nucleosome positioning and modifications in epigenetic gene regulation.

## Results

### Identification of positioned nucleosomes with epigenetic marks from ChIP-Seq

The dataset from Barski *et al. *contains one ChIP-Seq profile for each histone modification studied in CD4^+ ^human T cells, totaling 185.7 million 25 nt tags that are uniquely mapped to the human genome [16]. Despite Solexa technology's unique ability to sequence millions of tags from each sample, most locations in the human genome still lack enough coverage from a single sample alone to allow an accurate identification of positioned nucleosomes. To overcome this difficulty, we combined all 185 million ChIP-Seq tags in our search for positioned nucleosomes. Each Solexa sequencing tag of 25 nt represents one end of a ChIP-DNA fragment, corresponding to approximately 150 nt long mono-nucleosomal DNA resulting from MNase digestion. Therefore, we extended each sequenced tag to 150 nt in the 3' direction to represent a whole mono-nucleosome. This method of combining ChIP-Seq tags from 21 different histone modifications and extending each tag increased the original sequence data from individual sample by approximately 100-fold (approximately 21- and 6-folds from tag combination and extension, respectively) and provided over 10-fold non-redundant genome coverage (since all Solexa tags are uniquely mapped to the genome), sufficient to identify positioned nucleosomes.

**Figure 1:**
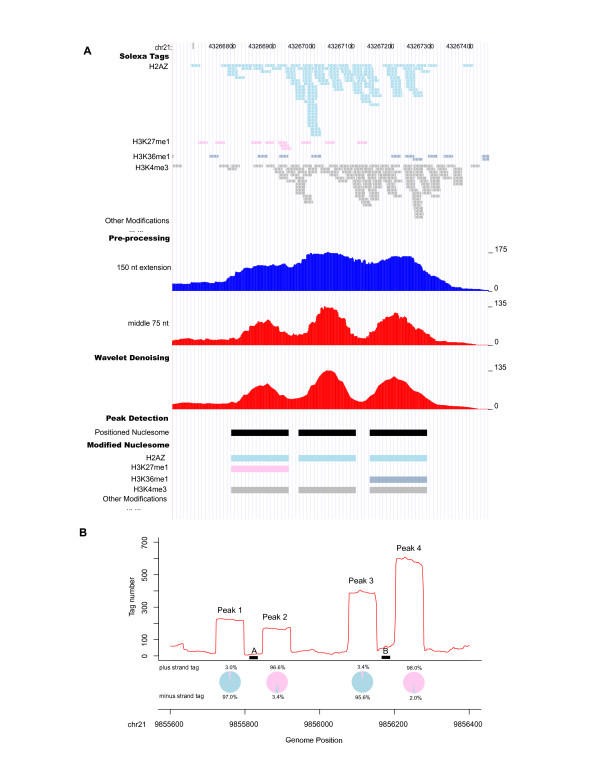
**Genome-wide identification of positioned nucleosomes from ChIP-Seq data**. (A) An example showing the workflow from sequencing tags to assigned modified nucleosomes: ChIP-Seq tags for all the available histone modifications in CD4^+ ^T cell (shown tags for four representative marks, with arrows in each indicating its strand orientation) are combined; each tag is extended towards 3' to 150 nt (legends on the right denote tag count); taking the middle 75 nt of all the extended tags provides better signal resolution; wavelet denoising smoothes the signal; LoG identifies peaks as positioned nucleosomes; histone modifications are assigned to individual identified nucleosomes. (B) Examples of peaks with unbalanced tag counts. Narrow regions A and B were repeatedly sequenced, creating the four flanking peaks with unbalanced tag counts between plus (pink) and minus (blue) strands as shown in the pie charts. They might arise from repetitive sequences in unsequenced or unannotated genome regions, thus were removed from further analysis.

The collection of extended sequence tags can be regarded as a random sampling of modified nucleosomes in the human genome. In this view, given the almost periodic nature of nucleosome locations, the length of each tag can be actually shortened based on the intuition offered by the Nyquist-Shannon sampling theorem [19] from signal processing. The theorem states that for a correct pattern reconstruction, the sampling frequency must be at least twice the maximum frequency of the signal of interest. In our context, the statement roughly implies that the tag length should be at most half the nucleosome length. Motivated by this principle, we took only the middle 75 nt sequences of all extended tags as inputs to our nucleosome identification algorithm. As shown in Figure 1A, keeping only the central 75 nt gave better resolution of nucleosome positioning than the full 150 nt. Using tag lengths shorter than 75 nt increases noise although it may give a sharper nucleosome positioning signal (Figure S1 in Additional file 1). After the tag extension, wavelet denoising was first applied to reduce the genomic background noise and smooth the sequencing tag counts across the genome. Laplacian of Gaussian (LoG) technique was then used to globally identify peaks of qualifying width (80–250 nt) as positioned nucleosomes (see Methods).

Most detected peaks had similar numbers of tags from plus and minus strands, since double stranded DNA fragments are equally likely to be sequenced from either direction. However, some peaks had significantly unbalanced tag counts (i.e., the number of tags from one strand was more than 4-fold higher than that from the other strand). Examples of this phenomenon are shown in Figure 1B, where hundreds of tags were aligned to very narrow regions A (27 nt) and B (33 nt). Extending the tags in the 3' direction to 150 nt and taking the middle 75 nt created two separate peaks flanking A (peaks 1 and 2) and B (peaks 3 and 4), thus creating unbalanced tag counts between plus and minus strands for those peaks. It is worth noting that both regions A ('ggtctagaatggaatggaaagaatgga') and B ('atacaatcgattggaatcgaatggaatggaagg') are near the centromere and both contain the repeated sequence 'tggaa', which is a type of common sequence repeats found near human centromeres [20]. Although the sequences of both regions A and B are uniquely mapped to the current version of human genome assembly (NCBI Build 36.1 or UCSC Hg18), it is possible that they occur in multiple copies near or within the centromeres, which may not have been yet fully sequenced and annotated. To avoid any complications arising from repeat sequences, we thus regarded the detected peaks with unbalanced tag counts as unreliable and discarded them from further analysis. This procedure removed 5% of the peaks, and the remaining 438,652 peaks were retained as confident positioned nucleosomes in CD4^+ ^T cells.

Based on the ChIP-Seq tags for individual histone methylations, we assigned histone methylation marks to each identified nucleosome (see Methods). 99.97% of the positioned nucleosomes had one or more kinds of histone modifications, with an average of 4.4 modifications per nucleosome. Table S1 in Additional file 1 listed the numbers of modified positioned nucleosomes for each histone modification. In Table S1 in Additional file 1, we also classified the 21 ChIP-Seq histone modifications into three types: active marks (related to active genes [16] or active enhancers [8]), repressive marks (related to inactive genes [16] or heterochromatin [21]), and moderate marks (no clear preference between active and inactive genes [16]). The genome locations of identified positioned nucleosomes and their histone modification profiles are available at .

### Distribution of modified positioned nucleosomes

**Figure 2:**
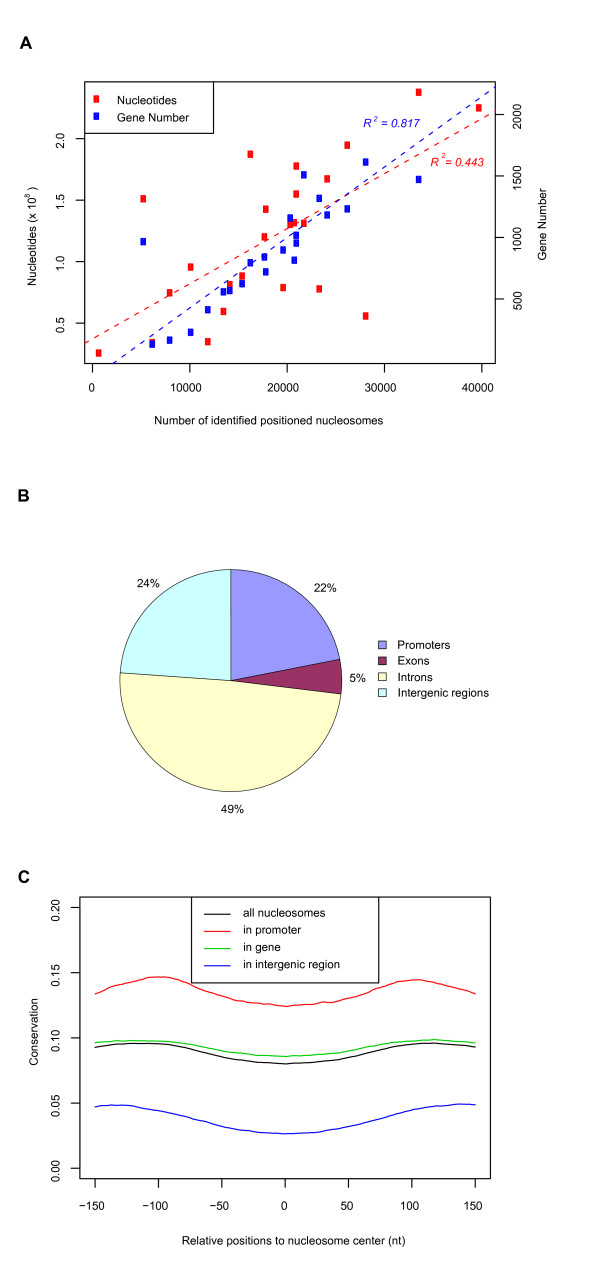
**Genomic distribution of identified positioned nucleosomes**. (A) Correlation of identified positioned nucleosomes on each chromosome with chromosome length and gene density. (B) Genomic distribution of identified positioned nucleosomes. (C) PhastCons conservation scores are lower at center-aligned positioned nucleosomes, and higher at flanking linker regions, regardless of whether nucleosomes occur in genes, promoters or intergenic regions.

While the identified positioned nucleosomes with histone modifications were modestly correlated with chromosome length (*R*^2 ^= 0.443, Figure 2A), they had a much stronger correlation with gene density (*R*^2 ^= 0.817, Figure 2A). This observation suggested biological functions of modified positioned nucleosomes in controlling gene activities. In addition, we found that 22% of the identified positioned nucleosomes appeared in promoters within 2.5 kb of transcription start sites (TSSs) – 6.8 times more than would be expected by random chance (~1 positioned nucleosomes per kb). Similarly, 54% were located in genes (defined as the region from 2.5 kb downstream of the TSS to the transcription end site, Figure 2B) – 1.6 times more than expected by chance. The remaining 24% of positioned nucleosomes often appeared far away from genes, with an average distance to the nearest TSS of 110 kb. Surprisingly, 76% of these distal positioned nucleosomes contained the transcriptional promoter signature H3K4me3 or enhancer signature H3K4me1/2 [8], indicating those nucleosomes may reside in functionally important regions and participate in the regulation of enhancers, repressors and insulators, or may lie near unannotated genes in the genome. In order to test the robustness of our observations, we used different p-value cutoffs for nucleosome identification. Although the number of identified nucleosomes varies with the cutoff, the general genomic distribution of identified nucleosomes is not affected (Table S2 and Figure S2 in Additional File 1).

Sequences in nucleosome free regions have been found to be more evolutionarily conserved than nucleosome-occupied regions in both yeast genome [10] and human promoters [12]. We calculated the average phastCons conservation scores [22] for all detected positioned nucleosomes in CD4^+ ^T cells and their nearby linker regions. Positioned nucleosomes in genes, promoters and intergenic sequences all have lower sequence conservation than their flanking linker DNA (Figure 2C). The linkers might be conserved because they harbor cis-acting elements to interact with trans-acting factors.

DNase I hypersensitive (HS) sites are regarded as containing a mixture of regulatory cis-elements. A recent study to map DNase I HS regions in CD4+ T cell discovered that only 2% of the genome is HS, with an average size of 634 nt [23]. We observed that 30% of our identified positioned nucleosomes were located in the DNase I HS regions. In addition, modified positioned nucleosomes were detected in 45% of all DNase HS regions, and in 99% of high confidence HS regions (DNase I HS quality score > 3, Figure S3 in Additional file 1). These observations suggest an epigenetic mechanism of modified positioned nucleosomes in establishing and maintaining functionally important regulatory regions in the human genome.

### Nucleosome function in gene regulation

**Figure 3:**
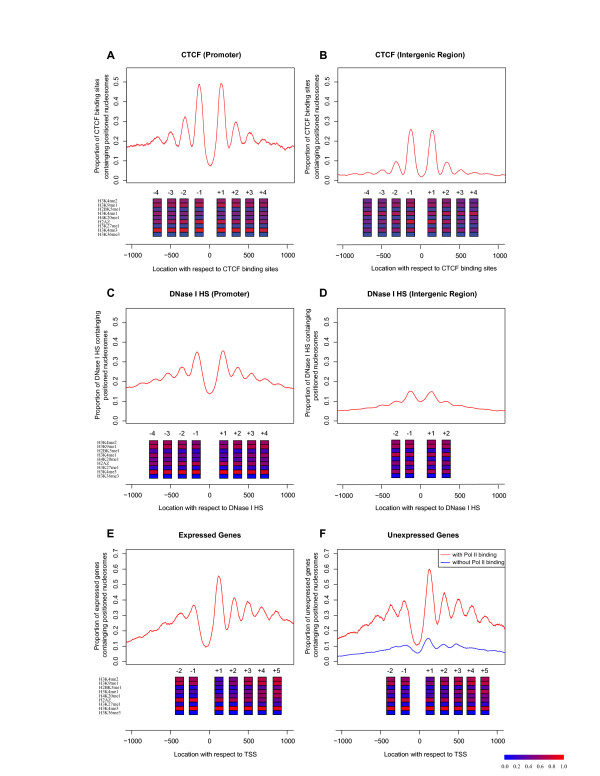
**Positioning and modification patterns of nucleosomes around cis-acting elements in CD4^+ ^T cell**. (A) CTCF binding sites at promoters and (B) intergenic regions, aligned by CTCF sequence motif (if present) or by peak-center (if absent). The y-axis denotes the average number of positioned nucleosomes for each binding site. Nine active histone modifications are ordered by ChIP-Seq efficiency as in Table S1 in Additional file 1, and shown at peak locations. Inactive marks are absent in positioned nucleosomes around CTCF binding sites, DNase I HS regions, and TSSs, thus are not drawn here. Colors indicate the proportion of positioned nucleosomes carrying a particular histone mark (key on the bottom). (C) DNase I HS sites in promoters and (D) in intergenic regions. (E) TSS of expressed genes and (F) unexpressed genes.

Nucleosomes are believed to play an important role in gene regulation by controlling the accessibility of DNA to trans-acting factors [2,24] and modulating the structure of chromatin [25]. We examined nucleosome positioning relative to the binding sites of CTCF [16], which is the only trans-acting factor (besides PolII) in human CD4^+ ^T cells with available genome-wide binding locations. Aligning the nucleosome data at CTCF sequence motifs if one was present [26] or at the centers of CTCF ChIP peaks otherwise, we observed strong positioned nucleosome patterns (Figure 3A–B). The center of CTCF binding showed strong depletion of nucleosomes, flanked by two equally well-positioned nucleosome peaks on both sides. Six out of the nine active marks were enriched in both flanking nucleosomes, suggesting a coordinated function of active histone marks to regulate nearby cis-regulatory sites. We observed stronger H3K4me3 at positioned nucleosomes near CTCF binding sites in promoters (Figure 3A) than in intergenic regions (Figure 3B), and the converse for H3K4me1, agreeing with known chromatin signatures of transcriptional promoters and enhancers [8], respectively. Our observation of nucleosome positioning and histone modification around CTCF binding sites is similar to a recent observation by Fu *et al. *[27]. In their paper, Fu *et al. *used the overall tag enrichment of nucleosome sequencing tags [17] around CTCF binding sites, while we took advantage of histone methylation ChIP-Seq data to infer positioned nucleosomes and then displayed the modification profile of identified positioned nucleosomes around CTCF binding sites.

DNase I HS sites are regarded as containing a mixture of regulatory cis-elements and are thus expected to exhibit nucleosome depletion similar to trans-acting factor binding sites. We examined the nucleosome profiles around DNase I HS sites [28] by aligning those regions by their peak summits, and we indeed found nucleosome depletion at the DNase I HS sites, flanked by two strong nucleosome positioning peaks (Figure 3C and 3D). Furthermore, while the nucleosome occupancy on both sides of DNase I HS sites is relatively lower than that of CTCF binding sites, the flanking nucleosomes showed similar histone modification profiles as in the case of CTCF binding regions.

There have been several studies using tiling arrays to investigate nucleosome positioning in yeast and human promoters [7,10-12]. Similarly, a recent study [29] revealed the promoter nucleosome architecture by separating the + and - strands of H3K4me3 and H2A.Z ChIP-Seq tags [16]. Here we examined the average number of positioned nucleosomes in the promoters of 4,517 expressed genes (Figure 3E) and 8,906 unexpressed genes (Figure 3F), obtaining much stronger nucleosome patterns than all previous studies in humans. For both expressed and unexpressed genes, the average number of positioned nucleosomes showed strong depletion approximately 50 nt upstream of TSS, although the depletion for unexpressed genes was weaker. The nucleosome signal gradually decreased as it oscillated away from TSS, but we could still clearly delineate five peaks downstream of TSS and at least two peaks upstream.

Detailed histone modification profiles on positioned nucleosomes near TSSs revealed interesting patterns, some of which could not be observed previously without the map of nucleosome-resolution modifications (Figure 3E). First of all, despite the fact that a decreasing number of genes contained a positioned nucleosome as the distance to TSS increased, the proportion of positioned nucleosomes carrying the H3K4me3 mark was constantly high. Three active marks (H3K4me1, H3K4me2, and H3K9me1) were rarely present at +1 nucleosome, but increased both upstream and downstream. Two other active marks H4K20me1 and H2BK5me1 were absent from upstream positioned nucleosomes, but showed increasing presence downstream from TSS. Since the function of H2BK5me1 mark was previously unknown and H4K20me1 was known as a transcription elongation mark in mammalian cells [30], we speculate that H2BK5me1 may also be a transcription elongation mark. In contrast, the active variant H2A.Z had high occupancy upstream and decreasing presence downstream. No H3K27me1, H3K36me3, repressive, or moderate marks appeared in positioned nucleosomes near TSS.

Surprisingly, these modification patterns were almost identical between expressed and unexpressed genes (Figure 3E–F). This observation suggests that a subset of genes with undetectable transcripts not only contain positioned nucleosomes near TSS, but also carry the identical histone modifications as expressed genes. Since the elongation mark H4K20me1 was present downstream of TSS and so was the proposed elongation mark H2BK5me1, despite being slightly weaker than in expressed genes, it was likely that these genes were actually transcribed but quickly degraded.

Several studies reported that certain histone modifications tend to occur in groups [7,9,14,31]. We also examined the histone modification co-occurrence patterns at nucleosome resolution, and we observed that active and repressive marks occurred mutually exclusively in the same nucleosomes (Figure S4 in Additional File 1). In order to test the robustness of our observations, we used different p-value cutoffs for assigning histone modifications. Although the number of nucleosomes with histone modifications varies, the general trend of correlation between histone modification profiles is not affected (Figure S5 in Additional File 1). We also found that adjacent nucleosomes avoided sharp transitions between active and repressive histone modifications and, instead, tended to carry the same or similar modifications (Figure S6 in Additional File 1). Our observations are supported by two recent publications [18,32]. Wang *et al. *combined the histone acetylation and methylation ChIP-Seq data to observe that certain histone modification marks tend to be grouped for activating gene expression [18]. Furthermore, Yu *et al. *used a Bayesian network to infer the relation between histone methylation marks and gene expression [32].

## Discussion

With the availability of commercial high-resolution tiling microarrays and high-throughput sequencing technologies, tremendous progress has been made towards understanding epigenetic regulation of gene expression. Nonetheless, studies on positioned nucleosomes with histone modification profiles at nucleosome resolution are still limited in human. We developed a signal-processing approach to identify positioned nucleosomes from existing histone methylation ChIP-Seq data and determined the histone modification states of each identified positioned nucleosome in CD4^+ ^human T cells.

**Figure 4:**
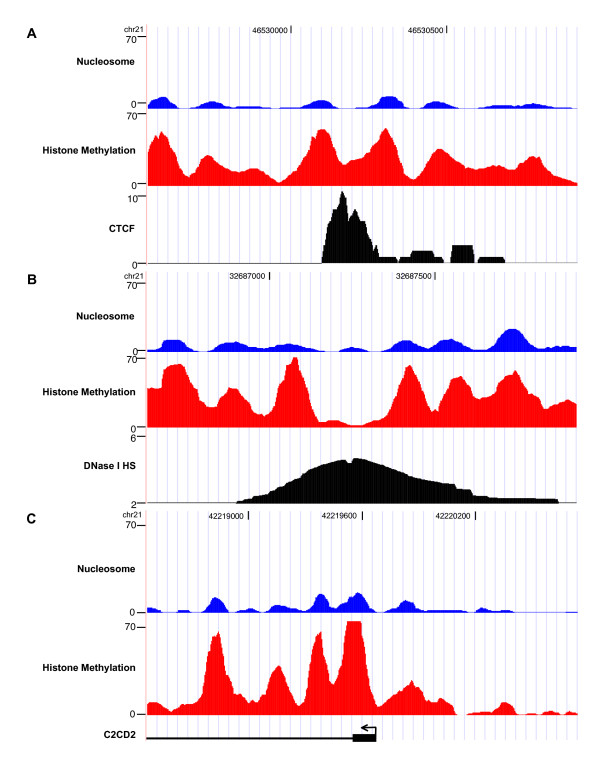
**Comparison of nucleosome positioning signals of histone methylation ChIP-Seq data and nucleosome sequencing data in functionally important regions**. Examples comparing nucleosome positioning signals from ChIP-Seq and nucleosome sequencing at regions around CTCF binding site (A), DNase I HS site (B) and TSS (C). The nucleosome positioning signal of methylation ChIP-Seq (combining 21 types of histone modifications) is shown in red color; while that of nucleosome sequencing is shown in blue color. Each tag is extended towards 3' to 150 nt and taken the middle 75 nt to generate the positioning signals; wavelet denoising was used to smooth the signals. The black profiles under the nucleosome positioning signals of methylation ChIP-Seq and nucleosome sequencing indicate a CTCF binding site (A), DNase I HS site (B), and the promoter of C2CD2 (C), respectively.

Despite the extensive scale and fine resolution of our analysis, our study did face potential biases and drawbacks. First, it will miss positioned nucleosomes that lack the particular set of histone modifications investigated by ChIP-Seq. In fact, we might even miss some modified nucleosomes if ChIP-Seq for the mark (e.g. repressive mark H3K9me3) is not sequenced deep enough. However, the identified positioned nucleosomes from ChIP-Seq appeared predominantly at functionally important regions. When compared with a recent study that sequenced over 150 million nucleosomes in CD4^+ ^T cells [17], our nucleosome positioning signals from ChIP-Seq data not only agreed with their results, but also yielded much stronger and distinct nucleosome patterns in functionally important regions (Figure 4), thus greatly facilitating the identification of positioned nucleosomes in such regions. Moreover, while nucleosome sequencing is becoming popular in smaller model organisms [33,34], the size of the mammalian genome requires extensive sequencing coverage at a substantial cost. Thus, our method of utilizing nucleosome-resolution histone modification ChIP-Seq data provides an alternative solution to identifying positioned nucleosomes in functionally important regions and, at the same time, can also provide information about the histone modification status of individual nucleosomes. As ChIP-Seq data on additional histone modifications under the same cell condition become available, this analysis could be repeated to generate an increasingly complete and accurate nucleosome map of functionally important regions.

Second, ChIP-Seq tags that were not uniquely mapped to the genome were discarded, so our approach will miss some regions with segmental duplication and repeat elements. Repeat sequences currently pose serious challenges to understanding the human genome and will require further novel advances in experimental and computational techniques. Third, the ChIP-Seq data [16] used in this study lacked a proper control sample, which ideally should be prepared from naked genomic DNA digested with MNase, size-selected and sequenced. Such a control sample could greatly benefit our analysis and improve the accuracy of our identified nucleosomes. For example, we could use it to eliminate false positives that might arise from unannotated repetitive sequences in the genome and Solexa sequencing bias (e.g. more tags in GC-rich regions). In addition, although we computationally calculated the false discovery rate (FDR) for positioned nucleosome identification from Poisson-based p-values, we could empirically estimate a more reasonable FDR of our approach that considers potential ChIP-Seq bias by comparing the numbers of peaks identified from ChIP and control.

## Conclusion

We developed a novel computational framework and efficiently identified over 438,000 positioned nucleosomes coupled with histone modifications in functionally important regions of the human genome. These nucleosomes are not evenly distributed across the genome: more than 75% are near genes, and the remaining ones far away from TSSs are likely around distal cis-regulatory elements such as enhancers and insulators. Our analysis of nucleosome positions and modifications near TSS, CTCF binding sites, and DNase I HS sites strongly support the dual role of nucleosomes in epigenetic regulation. First, chromatin structure at cis-regulatory elements may be controlled by histone modifications to recruit chromatin-associated proteins and maintain an open state, as supported by our analytical result that active and repressive marks do not occur together in the same or adjacent nucleosomes. Second, nucleosomes may be remodeled, exposing DNA binding sites to a variety of cellular machineries for transcription.

## Methods

The work flow is divided into two major components: nucleosome position detection and histone modification assignment to positioned nucleosomes (Figure 1).

### Nucleosome Position Detection

Nucleosome-level ChIP-Seq tags from all histone modifications were combined to provide enough genome coverage. Each 25 nt tag was extended to 150 nt in the 3' direction, and the middle 75 nt was used for nucleosome position detection. We then modified an approach that was applied to nucleosomes on tiling microarrays [12] to analyze the pre-processed data from ChIP-Seq. First, genomic regions with sparse tags (defined as fewer than 3 tags per kilobase (kb)) were discarded. The remaining regions were subjected to wavelet denoising to reduce the background noise in ChIP-Seq data. In this study, we chose coiflet4 as wavelet and scaling functions and performed decomposition at level two. Coiflet4 is suitable because its morphological characteristics are similar to the nucleosome peak shape [12]. Denoising at level two removed most high frequency noises, and further denoising appeared to deform the true peak shapes. We selected minimization of Stein's Unbiased Risk Estimate (SURE) and soft thresholding for estimating the threshold and filtering the signal, respectively [35,36]. SURE combined with soft thresholding is known to be suitable for denoising data containing edges (discontinuities), and matches well with our peak detection algorithm which perceives the edges as boundaries of positioned nucleosomes.

The peak detection algorithm calls peaks using LoG method, a combination of Gaussian window smoothing and Laplacian edge detection. Gaussian smoothing with an envelope size of 30 nt (which enabled us to achieve the best performance in peak detection in this study) was first adopted to further reduce the noise, then Laplacian edge detection was applied to find the inflection points of peaks in the data. The peaks detected by LoG were filtered based on the following criteria. First, the peak width should be between 80 and 250 nt. Second, the p-value of each peak was estimated as the probability that the same or larger number of tags can accumulate within the peak region by chance using Poisson approximation, and peaks with p-value greater than 10^-5 ^were discarded, which corresponds to 0.014% of FDR estimated by using a bootstrapping-based method [37] (Figure S7 in Additional file 1). Finally, for each peak, the number of tags from one strand should be less than four times that from the other strand.

### Histone modification assignment for nucleosomes

After positioned nucleosomes were detected as described above, the sequence tags were regrouped into different types of histone modification. The p-value of a particular histone modification at a positioned nucleosome was calculated based on the tag count of that histone modification in the nucleosome region using Poisson distribution, similar to the method mentioned above. A histone modification was assigned to the nucleosome if its tag count p-value in the region was less than 10^-3^.

### Dataset

The nucleosome-resolution (MNase digestion) ChIP-Seq Solexa tags for 20 types of histone methylation and histone variant H2A.Z as well as the ChIP-resolution (sonication) ChIP-Seq data for CTCF and Pol II in human CD4+ T cell [16] were downloaded from . The gene expression profiles of CD4+ T cell were obtained from SymAtlas [38]. CTCF ChIP peaks were detected using MACS [39].

### Software implementation

The software for positioned nucleosome identification from ChIP-Seq data is implemented in Python and publicly available at . The algorithm also works to detect positioned nucleosomes from nucleosome sequencing experiments (without ChIP). It requires two input files: a ChIP-Seq tags file (BED format) and a user-defined parameter file (TXT format). The users can easily set their own parameters other than the ones suggested above by editing the parameter file. The output of the software is a tab-delimited format file containing the chromosomal coordinates and p-values of the detected nucleosomes. In this work, the software processed 185 million Solexa tags within five hours on a Linux computer (CPU speed: 2 GHz).

## Abbreviations

ChIP: chromatin immunoprecipitation; LoG: Laplacian of Gaussian; TSS: transcription start sites; HS: hypersensitive; FDR: false discovery rate; SURE: Stein's Unbiased Risk Estimate.

## Authors' contributions

XSL, YZ and HS conceived the project and wrote the paper. YZ, HS and JSS designed the algorithm, performed the research and implemented the software, with the assistance of YL. All authors read and approved the final manuscript.

## Supplementary Material

Additional file 1 **Supplementary materials**. This file includes seven supplementary figures and two supplementary tables: Figure S1. Nucleosome positioning signal after extending each tag to 150 nt in the 3' direction and taking the middle 75 nt (A), 45 nt (B), and 25 nt (C). Figure S2. Genomic distribution of identified positioned nucleosomes under different p-value cutoff. Figure S3. Percentage of DNase I HS sites of human CD4^+ ^T cell containing identified positioned nucleosomes. Figure S4. Correlation between histone modification profiles at the same nucleosome loci. Figure S5. Co-occurrence of different histone modification pairs on the same positioned nucleosome loci under different cutoffs for histone modification assignment. Figure S6. Enrichment of co-occurrence of histone modification pairs on adjacent positioned nucleosome loci. Figure S7. Poisson-based p-values vs. FDR (q-value). Table S1. The ChIP-Seq tag number, modified nucleosome number, percentage of tags located in modified nucleosomes, ChIP-Seq efficiency and modification type for each histone modification. Table S2. Number and distribution of identified positioned nucleosomes under different p-value cutoff. Click here for file

## References

[B1] Luger K, Mader AW, Richmond RK, Sargent DF, Richmond TJ (1997). Crystal structure of the nucleosome core particle at 2.8 A resolution. Nature.

[B2] Lu Q, Wallrath LL, Elgin SC (1994). Nucleosome positioning and gene regulation. J Cell Biochem.

[B3] Workman JL, Kingston RE (1998). Alteration of nucleosome structure as a mechanism of transcriptional regulation. Annu Rev Biochem.

[B4] Luger K (2006). Dynamic nucleosomes. Chromosome Res.

[B5] Langst G, Becker PB (2004). Nucleosome remodeling: one mechanism, many phenomena?. Biochim Biophys Acta.

[B6] Rando OJ (2007). Global patterns of histone modifications. Curr Opin Genet Dev.

[B7] Liu CL, Kaplan T, Kim M, Buratowski S, Schreiber SL, Friedman N, Rando OJ (2005). Single-nucleosome mapping of histone modifications in S. cerevisiae. PLoS Biol.

[B8] Heintzman ND, Stuart RK, Hon G, Fu Y, Ching CW, Hawkins RD, Barrera LO, Van Calcar S, Qu C, Ching KA (2007). Distinct and predictive chromatin signatures of transcriptional promoters and enhancers in the human genome. Nat Genet.

[B9] Kurdistani SK, Tavazoie S, Grunstein M (2004). Mapping global histone acetylation patterns to gene expression. Cell.

[B10] Yuan GC, Liu YJ, Dion MF, Slack MD, Wu LF, Altschuler SJ, Rando OJ (2005). Genome-scale identification of nucleosome positions in S. cerevisiae. Science.

[B11] Mikkelsen TS, Ku M, Jaffe DB, Issac B, Lieberman E, Giannoukos G, Alvarez P, Brockman W, Kim TK, Koche RP (2007). Genome-wide maps of chromatin state in pluripotent and lineage-committed cells. Nature.

[B12] Ozsolak F, Song JS, Liu XS, Fisher DE (2007). High-throughput mapping of the chromatin structure of human promoters. Nat Biotechnol.

[B13] Pokholok DK, Harbison CT, Levine S, Cole M, Hannett NM, Lee TI, Bell GW, Walker K, Rolfe PA, Herbolsheimer E (2005). Genome-wide map of nucleosorne acetylation and methylation in yeast. Cell.

[B14] Bernstein BE, Kamal M, Lindblad-Toh K, Bekiranov S, Bailey DK, Huebert DJ, McMahon S, Karlsson EK, Kulbokas EJ, Gingeras TR (2005). Genomic maps and comparative analysis of histone modifications in human and mouse. Cell.

[B15] Bernstein BE, Humphrey EL, Erlich RL, Schneider R, Bouman P, Liu JS, Kouzarides T, Schreiber SL (2002). Methylation of histone H3 Lys 4 in coding regions of active genes. Proc Natl Acad Sci USA.

[B16] Barski A, Cuddapah S, Cui K, Roh TY, Schones DE, Wang Z, Wei G, Chepelev I, Zhao K (2007). High-resolution profiling of histone methylations in the human genome. Cell.

[B17] Schones DE, Cui K, Cuddapah S, Roh TY, Barski A, Wang Z, Wei G, Zhao K (2008). Dynamic regulation of nucleosome positioning in the human genome. Cell.

[B18] Wang Z, Zang C, Rosenfeld JA, Schones DE, Barski A, Cuddapah S, Cui K, Roh TY, Peng W, Zhang MQ (2008). Combinatorial patterns of histone acetylations and methylations in the human genome. Nat Genet.

[B19] Oppenheim AV, Schafer RW (1989). Prentice-Hall.

[B20] Prosser J, Frommer M, Paul C, Vincent PC (1986). Sequence relationships of three human satellite DNAs. J Mol Biol.

[B21] Kouzarides T (2007). Chromatin modifications and their function. Cell.

[B22] Siepel A, Bejerano G, Pedersen JS, Hinrichs AS, Hou M, Rosenbloom K, Clawson H, Spieth J, Hillier LW, Richards S (2005). Evolutionarily conserved elements in vertebrate, insect, worm, and yeast genomes. Genome Res.

[B23] Boyle AP, Davis S, Shulha HP, Meltzer P, Margulies EH, Weng Z, Furey TS, Crawford GE (2008). High-resolution mapping and characterization of open chromatin across the genome. Cell.

[B24] Anderson JD, Widom J (2000). Sequence and position-dependence of the equilibrium accessibility of nucleosomal DNA target sites. J Mol Biol.

[B25] Li B, Carey M, Workman JL (2007). The role of chromatin during transcription. Cell.

[B26] Kim TH, Abdullaev ZK, Smith AD, Ching KA, Loukinov DI, Green RD, Zhang MQ, Lobanenkov VV, Ren B (2007). Analysis of the vertebrate insulator protein CTCF-binding sites in the human genome. Cell.

[B27] Fu Y, Sinha M, Peterson C, Weng Z, Van Steensel B (2008). The Insulator Binding Protein CTCF Positions 20 Nucleosomes around Its Binding Sites across the Human Genome. PLoS Genet.

[B28] Boyle AP, Davis S, Shulha HP, Meltzer P, Margulies EH, Weng Z, Furey TS, Crawford G (2008). High-resolution mapping and characterization of open chromatin across the genome. Cell.

[B29] Schmid CD, Bucher P (2007). ChIP-Seq Data Reveal Nucleosome Architecture of Human Promoters. Cell.

[B30] Vakoc CR, Sachdeva MM, Wang H, Blobel GA (2006). Profile of histone lysine methylation across transcribed mammalian chromatin. Mol Cell Biol.

[B31] Schubeler D, MacAlpine DM, Scalzo D, Wirbelauer C, Kooperberg C, van Leeuwen F, Gottschling DE, O'Neill LP, Turner BM, Delrow J (2004). The histone modification pattern of active genes revealed through genome-wide chromatin analysis of a higher eukaryote. Genes Dev.

[B32] Yu H, Zhu S, Zhou B, Xue H, Han JD (2008). Inferring causal relationships among different histone modifications and gene expression. Genome Research.

[B33] Valouev A, Ichikawa J, Tonthat T, Stuart J, Ranade S, Peckham H, Zeng K, Malek JA, Costa G, McKernan K (2008). A high-resolution, nucleosome position map of C. elegans reveals a lack of universal sequence-dictated positioning. Genome Res.

[B34] Shivaswamy S, Iyer VR (2008). Stress-dependent dynamics of global chromatin remodeling in yeast: dual role for SWI/SNF in the heat shock stress response. Mol Cell Biol.

[B35] Donoho DL, Johnstone IM (1995). Adapting to Unknown Smoothness via Wavelet Shrinkage. Journal of the American Statistical Association.

[B36] Donoho DL (1995). De-noising by soft-thresholding. Information Theory, IEEE Transactions on.

[B37] Storey JD, Tibshirani R (2003). Statistical significance for genomewide studies. Proc Natl Acad Sci USA.

[B38] Su AI, Wiltshire T, Batalov S, Lapp H, Ching KA, Block D, Zhang J, Soden R, Hayakawa M, Kreiman G (2004). A gene atlas of the mouse and human protein-encoding transcriptomes. Proc Natl Acad Sci USA.

[B39] Zhang Y, Liu T, Meyer CA, Eeckhoute J, Johnson DS, Bernstein BE, Nussbaum C, Myers RM, Brown M, Li W (2008). Model-based Analysis of ChIP-Seq (MACS). Genome Biol.

