# Supplementary material for: Identifying Positioned Nucleosomes with Epigenetic Marks in Human from ChIP-Seq
Source: BMC Genomics. 2008 Nov 13;9:537. doi: 10.1186/1471-2164-9-537 (PMC2596141; doi:10.1186/1471-2164-9-537)
Supplement: Additional file 1 — Supplementary materials. This file includes seven supplementary figures and two supplementary tables: Figure S1. Nucleosome positioning signal after extending each tag to 150 nt in the 3' direction and taking the middle 75 nt (A), 45 nt (B), and 25 nt (C). Figure S2. Genomic distribution of identified positioned nucleosomes under different p-value cutoff. Figure S3. Percentage of DNase I HS sites of human CD4+ T cell containing identified positioned nucleosomes. Figure S4. Correlation between histone modification profiles at the same nucleosome loci. Figure S5. Co-occurrence of different histone modification pairs on the same positioned nucleosome loci under different cutoffs for histone modification assignment. Figure S6. Enrichment of co-occurrence of histone modification pairs on adjacent positioned nucleosome loci. Figure S7. Poisson-based p-values vs. FDR (q-value). Table S1. The ChIP-Seq tag number, modified nucleosome number, percentage of tags located in modified nucleosomes, ChIP-Seq efficiency and modification type for each histone modification. Table S2. Number and distribution of identified positioned nucleosomes under different p-value cutoff. [file 1471-2164-9-537-S1.pdf]

Supplementary Figures

Figure S1. Nucleosome positioning signal after extending each tag to 150 nt in the 3' direction and extracting the middle 75 nt (A), 45 nt (B), and 25 nt (C).

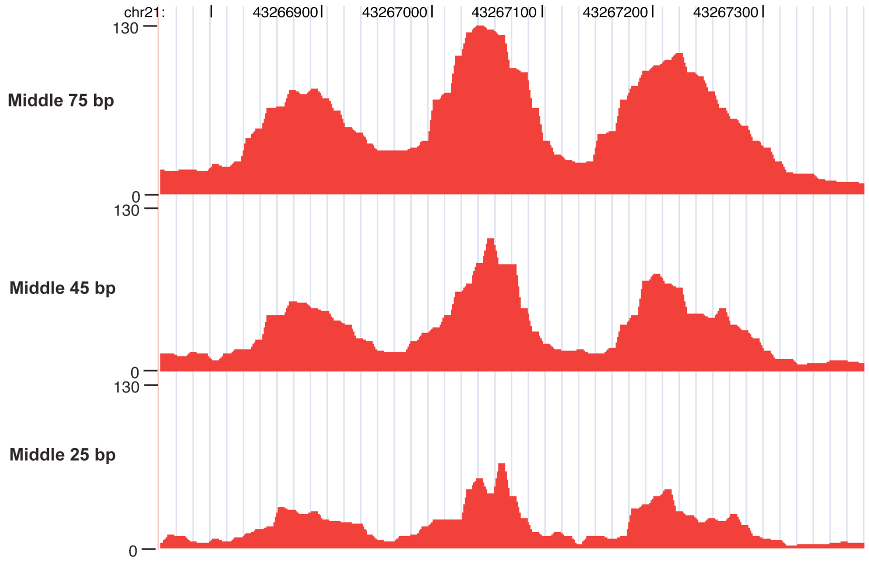

Figure S2. Genomic distribution of identified positioned nucleosomes under different p-value cutoffs.

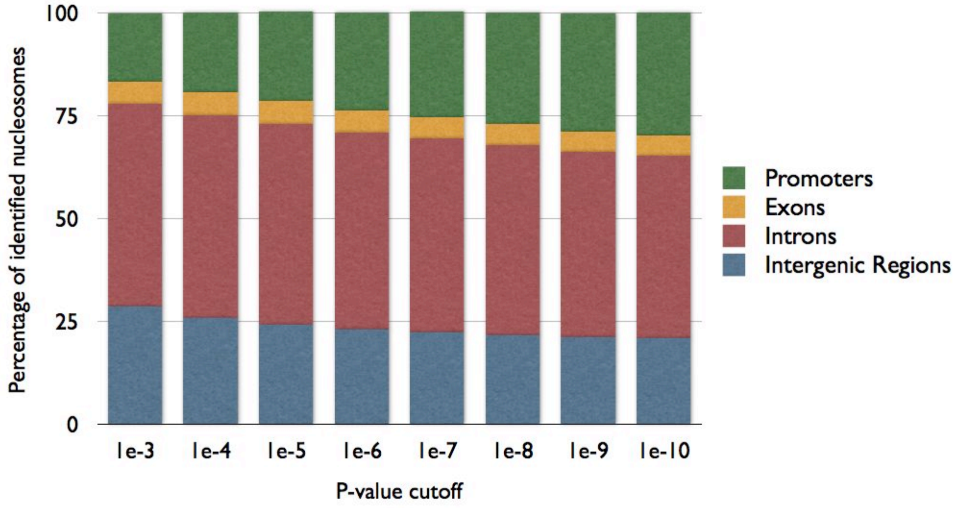

Figure S3. Percentage of DNase I hypersensitive sites of human CD4^{+} T cell containing identified positioned nucleosomes.

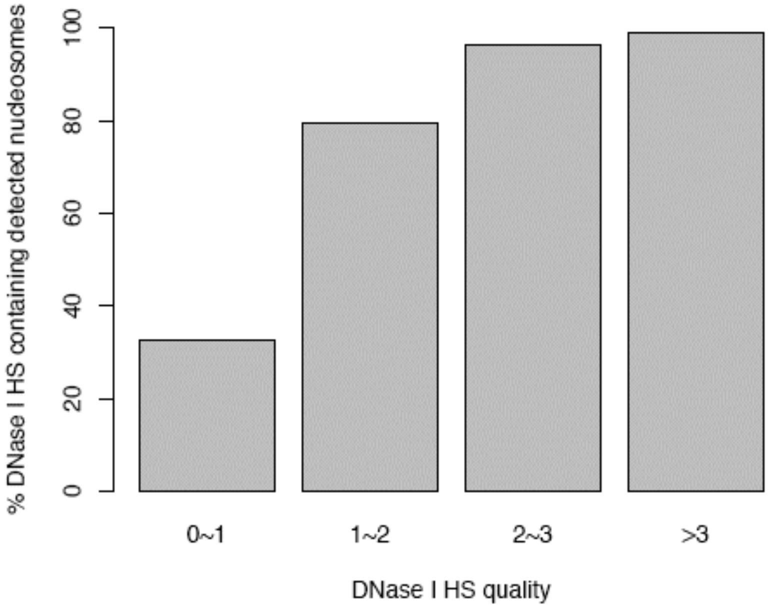

Figure S4. Co-occurrence of different histone modification pairs on the same positioned nucleosome loci. Histone modifications are ordered by ChIP-Seq efficiency as in **Table S1 in Additional file 1**. Colors show the $\log_2$ ratio of observed over expected co-occurrence rates of each pair of the histone modifications. The expected co-occurrence rate of any pair of histone modifications was estimated based on the assumption that the two occurred independently in the genome.

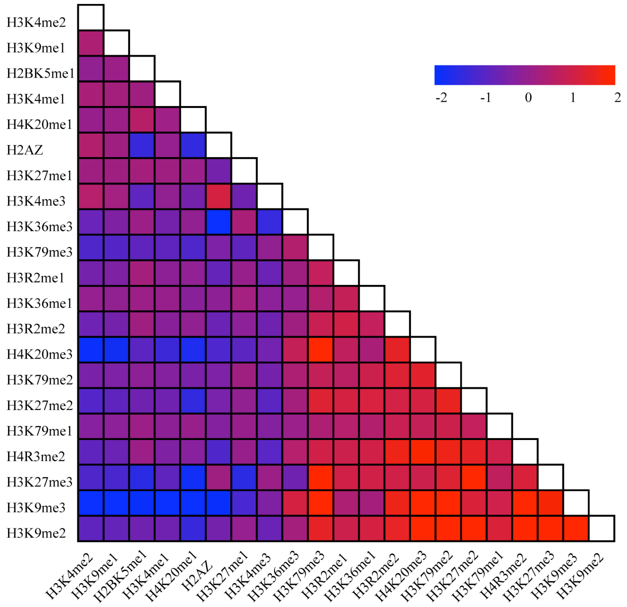

Figure S5. Co-occurrence of different histone modification pairs on the same positioned nucleosome loci. The p-value cutoffs for assigning modifications are (A) 10^{-5} and (B) 10^{-10}.

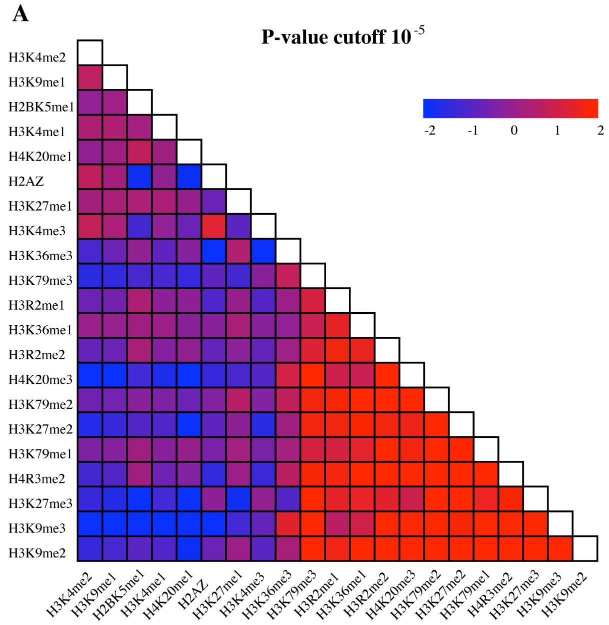

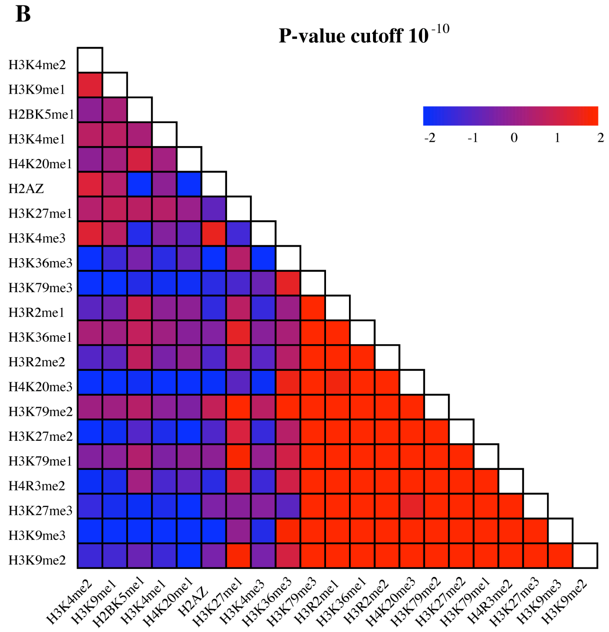

Figure S6. Enrichment of co-occurrence of histone modification pairs on adjacent positioned nucleosome loci. (A) Colors show the $\log_2$ ratio of observed over expected co-occurrence rates of each pair of the histone modifications. Totally, 114,749 adjacent nucleosome pairs were chosen, where the maximum allowed distance between the centers of two adjacent nucleosomes was 250 nt. **(B)** Colors show the p-value for the enrichment of pairs of histone modifications on adjacent nucleosome loci.

A

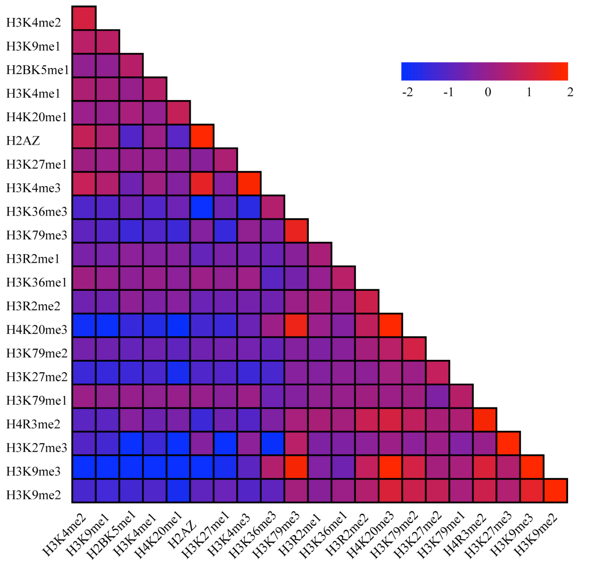

-2
-1
0
1
2
H3K4me2
H3K9me1
H2BK5me1
H3K4me1
H4K20me1
H2AZ
H3K27me1
H3K4me3
H3K36me3
H3K79me3
H3R2me1
H3K36me1
H3R2me2
H4K20me3
H3K79me2
H3K27me2
H3K79me1
H4R3me2
H3K27me3
H3K9me3
H3K9me2

B

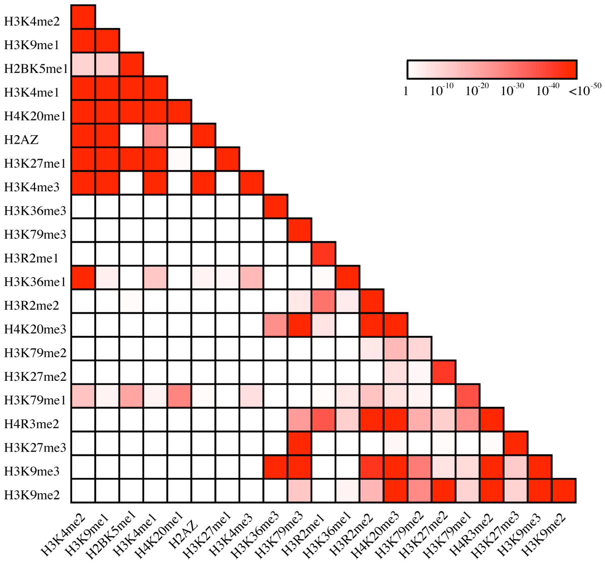

1
10^{-10}
10^{-20}
10^{-30}
10^{-40}
$<10^{-50}$
H3K4me2
H3K9me1
H2BK5me1
H3K4me1
H4K20me1
H2AZ
H3K27me1
H3K4me3
H3K36me3
H3K79me3
H3R2me1
H3K36me1
H3R2me2
H4K20me3
H3K79me2
H3K27me2
H3K79me1
H4R3me2
H3K27me3
H3K9me3
H3K9me2

Figure S7. Poisson-based p-values vs. FDR (q-value). The FDR was estimated by applying Storey *et al.*'s method [1] to the p-values of positioned nucleosomes identified by NPS. The vertical dashed-line indicates the p-value cutoff of 10^{-5} that we used in our study and corresponds to a 0.014% FDR.

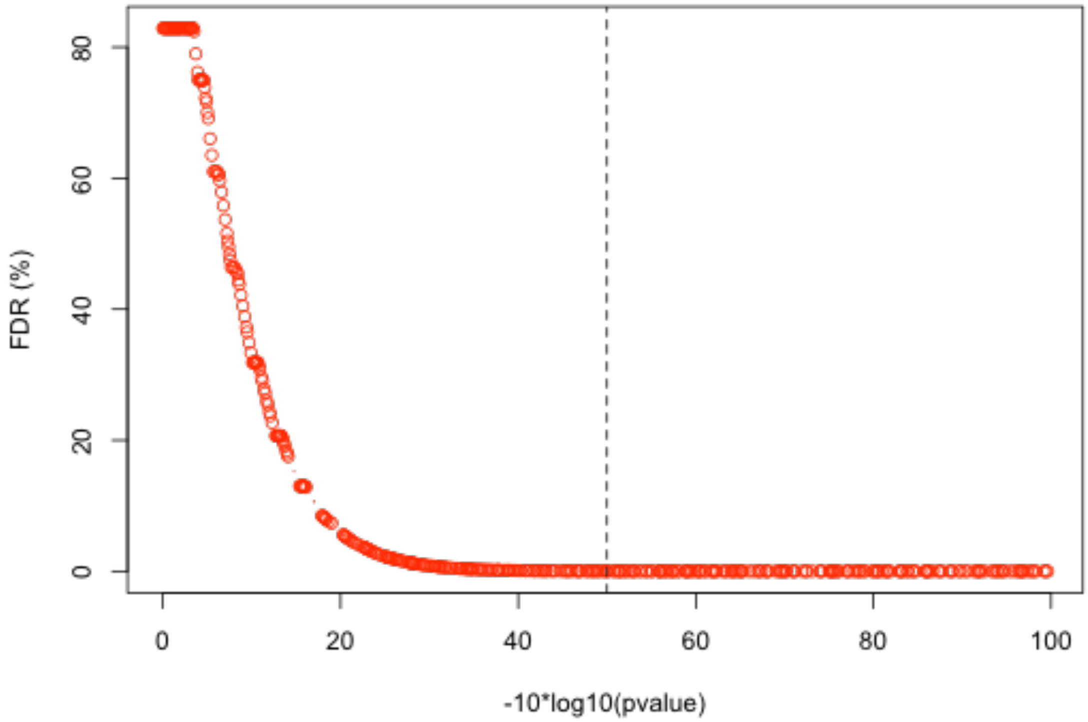

1. Storey JD, Tibshirani R: **Statistical significance for genomewide studies**. *Proc Natl Acad Sci USA* 2003, **100**(16):9440-9445.

Supplementary Tables

Table S1. Summary statistics for histone modifications.

Histone Modification	Aligned ChIP-Seq Tag Number	Modified Nucleosome Num	% Tags in Modified Nucleosomes	ChIP-Seq Efficiency[1]	Modification Type[2]
H3K4me2	5,447,902	183,363	23.23	33,658	A
H3K9me1	9,311,627	251,219	20.20	26,979	A
H2BK5me1	8,942,880	205,864	22.34	23,020	A
H3K4me1	11,322,526	254,748	31.26	22,499	A
H4K20me1	11,015,873	209,660	28.10	19,033	A
H2AZ	7,536,100	108,198	19.16	14,357	A
H3K27me1	10,047,279	140,633	7.30	13,997	A
H3K4me3	16,845,478	153,220	35.46	9,096	A
H3K36me3	13,572,575	120,050	9.14	8,845	A
H3K79me3	8,114,474	53,177	4.16	6,553	M
H3R2me1	9,560,224	61,340	2.92	6,416	M
H3K36me1	8,077,127	41,080	1.95	5,086	M
H3R2me2	5,720,089	22,468	8.15	3,928	M
H4K20me3	6,521,560	22,291	1.58	3,418	M
H3K79me2	2,058,068	6,295	0.81	3,059	M
H3K27me2	9,070,882	24,619	1.19	2,714	M
H3K79me1	10,041,806	26,267	0.97	2,616	M
H4R3me2	7,357,597	19,234	1.24	2,614	M
H3K27me3	8,970,141	18,405	0.99	2,052	R
H3K9me3	6,348,997	10,726	1.15	1,689	R
H3K9me2	9,782,127	11,969	0.54	1,224	R

[1] ChIP-Seq efficiency is defined as the number of identified modified nucleosomes per million tags.
[2] A: active mark; R: repressive mark, M: moderate mark.

Table S2. Number and distribution of identified positioned nucleosomes under different p-value cutoffs. The 3rd and 4th columns are the R-squared for the linear model to show the correlation of identified positioned nucleosomes on each chromosome with gene density (gene numbers) and chromosome length (nucleotides).

P-value cutoff	Number of identified positioned nucleosome	R-squared	
		Gene numbers	Nucleotides
10^{-3}	682,080	0.771	0.544
10^{-4}	533,224	0.799	0.487
10^{-5}	438,652	0.817	0.443
10^{-6}	379,271	0.826	0.411
10^{-7}	332,309	0.832	0.384
10^{-8}	301,098	0.834	0.364
10^{-9}	269,688	0.834	0.343
10^{-10}	249,734	0.835	0.331
